# Fiberoptic endoscopic evaluation of swallowing in amyotrophic lateral sclerosis: comparison with older people with dysphagia and relationship with time since diagnosis

**DOI:** 10.1590/2317-1782/e20240296en

**Published:** 2025-11-17

**Authors:** Ramon Cipriano Pacheco de Araújo, Cynthia Meira de Almeida Godoy, Lidiane Maria de Brito Macedo Ferreira, Juliana Fernandes Godoy, Hipólito Magalhães

**Affiliations:** 1 Programa Associado de Pós-graduação em Fonoaudiologia – PPGFON, Universidade Federal do Rio Grande do Norte – UFRN - Natal (RN), Brasil.; 2 Hospital Universitário Onofre Lopes – HUOL - Natal (RN), Brasil.; 3 Departamento de Fonoaudiologia, Universidade Federal do Rio Grande do Norte – UFRN - Natal (RN), Brasil.

**Keywords:** Amyotrophic Lateral Sclerosis, Deglutition Disorders, Deglutition, Aged, Pharynx

## Abstract

**Purpose:**

(1) to compare the findings of the instrumental swallowing assessment between individuals with amyotrophic lateral sclerosis (ALS) and older dysphagic adults without neurological diagnosis; (2) to compare the onset of pharyngeal response, pharyngeal residues, and the level of oral intake in relation to the time since diagnosis in the ALS group.

**Methods:**

This cross-sectional, retrospective study collected data from medical records. Altogether, 101 individuals with dysphagia were included and stratified into two groups: the first had 56 patients diagnosed with ALS, and the second had 45 older adults. Dysphagia signs were analyzed through fiberoptic endoscopic evaluation of swallowing, using four food consistencies, classified by the International Dysphagia Diet Standardisation Initiative (IDDSI). Pharyngeal residues were classified by the Yale Pharyngeal Residue Severity Rating Scale (YPRSRS), and oral intake by the Functional Oral Intake Scale (FOIS).

**Results:**

The ALS group had differences in multiple swallows with one IDDSI consistency; posterior oral leakage, pharyngeal residues, and laryngeal penetration with three consistencies; and aspiration with one consistency. Individuals with more than 3 years since diagnosis had differences in the onset of the pharyngeal response in the pyriform sinuses, moderate pharyngeal residues, and oral intake.

**Conclusion:**

The ALS group had significant differences in the occurrence of multiple swallows, posterior oral leakage, pharyngeal residues, penetration, and aspiration with three IDDSI consistencies. Furthermore, the time since diagnosis was a determining factor for all three parameters.

## INTRODUCTION

Amyotrophic lateral sclerosis (ALS) is a progressive neurodegenerative disease that affects upper and lower motor neurons, often manifesting in adulthood^(1)^. Its etiopathogenesis involves degeneration of the motor system at different levels (e.g., bulbar, cervical, thoracic, and limb), severely affecting quality of life, especially functioning and motor capacity^(2)^. One of the most common symptoms in early stages is difficulty swallowing, which progresses to a debilitating stage^(3)^. As the disease progresses, respiratory complications, such as aspiration pneumonia resulting from severe oropharyngeal dysphagia, can be the cause of death^(4)^.

Dysphagia can affect all phases of swallowing in individuals with ALS, progressing to the complete inability to swallow orally^(2)^. Evidence indicates progressive weakening of the oropharyngeal muscles, especially the tongue, as well as reduced expiratory airflow, such as cough flow, which is essential for protecting the lower airways after swallowing^(5)^. Approximately 30% of patients with bulbar symptoms develop speech and swallowing disorders in the early stages of the disease, with progressive worsening over the clinical course^(6)^. Advanced age and longer time since diagnosis are associated with more severe dysphagia^(7)^.

The aging process is associated with several physiological changes, including neurological deterioration and loss of muscle mass in specific regions, with a negative impact on swallowing^(8)^. Decreased muscle mass, strength, and elasticity of oropharyngeal connective tissues result in reduced functional efficiency of the tongue, lips, and jaw^(9)^. Older adults have accumulated adipose tissue and increased deposition of connective tissue^(8)^. In general, these changes contribute to reduced effectiveness of the chewing and swallowing mechanisms^(10)^. Decreased elasticity of connective tissues can restrict anterior laryngeal movement, increasing the risk of pharyngeal residue after swallowing^(11)^. These changes, intrinsically related to aging, increase the susceptibility to dysphagia^(10,11)^. Furthermore, additional physiological stressors, such as hospitalizations or adverse drug effects, can further compromise the balance between swallowing changes and the person's compensatory capacity^(11)^.

According to current epidemiological data, the mean age of onset of sporadic ALS is approximately 60 years^(12)^. However, several population-based studies have reported a progressive increase in the mean age of onset, as observed in Europe, where the mean is 63.7 years^(13,14)^. Furthermore, there is growing evidence of an increase in the incidence rate of ALS with advancing age, with a higher prevalence in older individuals^(15)^. Considering that these findings reflect a higher concentration of the disease in older people, there is increasing interest in investigating and identifying possible differences in the pathophysiology of swallowing between individuals with ALS and older dysphagic individuals without a neurological diagnosis.

Thus, this study aimed to compare swallowing parameters between individuals with ALS and older individuals without neurological diagnosis and compare the onset of pharyngeal response, pharyngeal residues, and the level of oral intake in relation to the time since diagnosis in the ALS group.

## METHODS

This is a cross-sectional, retrospective study based on data collected from medical records. The research was conducted at the otorhinolaryngology outpatient clinic of Onofre Lopes University Hospital, Federal University of Rio Grande do Norte, Natal, Brazil. Data were collected from fiberoptic endoscopic evaluation of swallowing (FEES) of patients treated between 2017 and 2023. All participants or their legal guardians signed an informed consent form provided by the service before the examination procedures. The study was approved by the Research Ethics Committee of Onofre Lopes University Hospital, under approval no. 6,169,294. The data collected referred to the findings of the instrumental examination and the level of oral intake after the examination.

### Sample

The sample consisted of 101 dysphagic patients chosen by convenience from among those seeking care at the said facility. For the first objective, participants were divided into two groups according to the presence of an ALS diagnosis. The first group consisted of 56 individuals with ALS, regardless of the time since diagnosis, aged 39 to 83 years, predominantly males (57.1%). The second group consisted of 45 older adults, aged 60 to 90 years, with clinical complaints of dysphagia, without a diagnosis of neurological disease, predominantly females (57.7%).

For the second objective of the study, the ALS group was divided into two subgroups: “up to 3 years” and “more than 3 years” of diagnosis, for intragroup analysis. The cutoff point of 3 years was defined based on the literature, which indicates this period as the average for the introduction of enteral feeding in this population^(16)^.

The exclusion criteria for both groups were (a) other acute or progressive neurological diagnoses; (b) inability to follow commands; (c) history of head and neck cancer treatment; (d) tracheostomy users; (e) history of orotracheal intubation in the last 12 months before the examination.

We emphasize that all research participants had swallowing complaints and were undergoing investigation of dysphagia, identified by other health professionals and/or referred by other hospital sectors in shared medical appointments.

### Procedures

FEES was performed by a resident physician, accompanied by an otolaryngologist and a speech-language-hearing pathologist with experience in oropharyngeal dysphagia, in accordance with the institution's protocol. They used a flexible Olympus® nasofibroscope, 3.2 mm in diameter, model LF-P, with an attached microcamera and light source. The patient was instructed to remain seated and upright throughout the examination. No topical anesthetic was used during the insertion of the instrument into the nasal cavity down to the hypopharynx.

During inspection of the structures, pharyngeal sensitivity was assessed by means of two touches of the nasofibroscope in the epiglottic region, verifying the presence of a pharyngeal constriction reaction. Glottic closure was analyzed by asking the patient to emit a prolonged vowel “i”. Closure was considered reduced when the vocal folds closed in less than 2/3 of their length^(17)^.

The speech-language-hearing pathologist provided the liquids, which were free of artificial flavors, artificially colored with aniline blue, and thickened with an instant cornstarch product. At the end, an 8-g portion of saltine crackers was also offered on demand.

Food consistencies were classified according to the International Dysphagia Diet Standardisation Initiative (IDDSI)^(18)^ and followed the order of offering: level 2 (mildly thick liquid), level 4 (extremely thick liquid), and level 0 (thin liquid) in three offerings with a 5-mL metal spoon; level 7 (regular solid) was a single portion.

The three professionals mentioned above, with experience in performing the examination, interpreted and simultaneously evaluated by consensus the presence of multiple swallows, posterior oral leakage, pharyngeal residues in the valleculae and/or pyriform sinuses, according to the Yale Pharyngeal Residue Severity Rating Scale (YPRSRS)^(19)^ classification (1 – No residue, 2 - Pharyngeal residue, 3 - Mild residue, 4 - Moderate residue, 5 - Severe residue). They also evaluated laryngeal penetration and laryngotracheal aspiration.

The analyses considered the following parameters, based on the worst performance observed after the first offer: multiple swallows, considered as more than two attempts to swallow the same offer^(20)^; posterior oral leakage, due to the presence of premature leakage of food in the hypopharynx region before triggering the swallowing reaction^(20)^; pharyngeal residue, through the identification of residual presence of colored food in the region of valleculae and/or pyriform sinuses, after swallowing the first offer onwards^(19)^; laryngeal penetration, via observation of residual presence of colored food in the region of the vocal folds^(21)^; and laryngotracheal aspiration, when there was residue of colored food below the vocal folds^(21)^. All analyses occurred in real time, and the images were stored on a computer at the clinic to be reviewed as many times as the professionals deemed necessary after the examination.

The Functional Oral Intake Scale (FOIS)^(22)^ was applied after the examination to determine oral intake. The three professionals considered the instrumental findings, clinical history, and the presence and need for liquid thickening. FOIS scores range from 1 (no oral intake) to 7 (full oral intake without restrictions).

### Data analysis

Descriptive and inferential statistics were used for data analysis, using measures of central tendency and dispersion, as well as absolute and relative frequencies. The Shapiro-Wilk normality test was applied to verify the distribution of the study's dependent quantitative variables. Inferential analysis used Pearson's chi-square or Fisher's exact test to analyze categorical variables, depending on the expected frequency of each cell being greater than or equal to 5. Student's t-test was used to analyze numerical variables. The Mann-Whitney U test was used to analyze and compare the protocols after verifying normal distribution. A 5% significance level was considered for all analyses.

## RESULTS

The sample consisted of 101 dysphagic individuals divided into two groups according to the presence of an ALS diagnosis. The first group had 56 individuals diagnosed with ALS, 83.9% with sporadic ALS, and 16.0% with familial ALS. The second group had 45 older adults with dysphagia without a neurological diagnosis.

Among the older people, approximately 40% had comorbidities such as diabetes mellitus, 24.4% cardiovascular disease, 15.5% digestive diseases, and 8.8% depressive disorders. None of the participants had previously received speech-language-hearing therapy. Table 1 shows the comparative analysis between sex and age groups.

**Table 1:** Comparative analysis of sex and age between groups

Variables	Groups		p-value
	ALS	Older adults	
	n = 56 (%)	n = 45 (%)	
Sex			
Males	32 (57.1)	19 (42.2)	0.136
Females	24 (42.9)	26 (57.8)	
Age (years)	62.1 (± 11.4)	71.0 (± 7.72)	<0.001^*^

* Student's t-test

**Caption:** ALS = amyotrophic lateral sclerosis

Table 2 compares the instrumental examination findings by food consistency level between the groups. There were significant differences between all findings with thin liquid (level 0) and between posterior oral leakage, pharyngeal residue, and laryngeal penetration with mildly thick liquid (level 2) and extremely thick liquid (level 4). No differences were observed with regular solid food (level 7) between the groups.

**Table 2:** Comparison of fiberoptic endoscopic findings by food consistency between groups

Fiberoptic endoscopic findings by food consistency (IDDSI)	Groups		p-value
	ALS	Older adults	
	n = 56 (%)	n = 45 (%)	
Pharyngeal sensitivity			
Preserved	48 (85.7)	35 (77.8)	0.3
Reduced	8 (14.3)	10 (22.2)	
Glottal closure			
Preserved	53 (94.6)	44 (97.8)	0.422
Reduced	3 (5.4)	1 (2.2)	
Thin liquid (level 0)			
Multiple swallowing			
Absent	46 (82.1)	43 (95.6)	0.038**
Present	10 (17.9)	2 (4.4)	
Posterior oral leakage			
Absent	20 (35.7)	27 (60.0)	0.015*
Present	36 (64.3)	18 (40.0)	
Pharyngeal residues			
Absent	14 (25.0)	25 (55.6)	0.002^*^
Present	42 (75.0)	20 (44.4)	
Laryngeal penetration			
Absent	41 (73.2)	44 (97.8)	<0.001^**^
Present	15 (26.8)	1 (2.2)	
Laryngotracheal aspiration			
Absent	45 (80.4)	44 (97.8)	0.011**
Present	11 (19.6)	1 (2.2)	
Mildly thick liquid (level 2)			
Multiple swallowing			
Absent	48 (85.7)	43 (95.6)	0.178
Present	8 (14.3)	2 (4.4)	
Posterior oral leakage			
Absent	26 (46.4)	34 (75.6)	0.003*
Present	30 (53.6)	11 (24.4)	
Pharyngeal residues			
Absent	16 (28.6)	26 (57.8)	0.003*
Present	40 (71.4)	19 (42.2)	
Laryngeal penetration			
Absent	46 (82.1)	44 (97.8)	0.021**
Present	10 (17.9)	1 (2.2)	
Laryngotracheal aspiration			
Absent	51 (91.1)	45 (100)	0.064
Present	5 (8.9)	0 (0.0)	
Extremely thick liquid (level 4)			
Multiple swallowing			
Absent	46 (82.1)	43 (95.6)	0.061
Present	10 (17.9)	2 (4.4)	
Posterior oral leakage			
Absent	27 (48.2)	31 (68.9)	0.037*
Present	29 (51.8)	14 (31.1)	
Pharyngeal residues			
Absent	18 (32.1)	25 (55.6)	0.018*
Present	38 (67.9)	20 (44.4)	
Laryngeal penetration			
Absent	50 (89.3)	45 (100)	0.032**
Present	6 (10.7)	0 (0.0)	
Laryngotracheal aspiration			
Absent	53 (94.6)	45 (100)	0.251
Present	3 (5.4)	0 (0.0)	
Regular solid food (level 7)			
Multiple swallowing			
Absent	53 (94.6)	45 (100)	0.251
Present	3 (5.4)	0 (0.0)	
Posterior oral leakage			
Absent	49 (87.5)	40 (88.9)	0.83
Present	7 (12.5)	5 (11.1)	
Pharyngeal residues			
Absent	40 (71.4)	39 (86.7)	0.065
Present	16 (28.6)	6 (13.3)	
Laryngeal penetration			
Absent	56 (100)	45 (100)	-
Present	0 (0.0)	0 (0.0)	
Laryngotracheal aspiration			
Absent	56 (100)	45 (100)	-
Present	0 (0.0)	0 (0.0)	

All data were expressed as numbers (%)

* Pearson's chi-square test;

** Fisher's exact test

**Caption:** ALS = amyotrophic lateral sclerosis; IDDSI = International Dysphagia Diet Standardisation Initiative

Furthermore, there was a significant difference in the onset of pharyngeal response, severity of pharyngeal residues, and level of oral intake between the groups presented in Table 3. For the most part, the group with ALS presented onset of pharyngeal response in valleculae, pharyngeal residues classified as mild (YPRSRS - 3), and total oral intake with multiple consistencies, but requiring special preparation or compensations (FOIS 5).

**Table 3:** Comparison of the onset of pharyngeal response, severity of pharyngeal residues, and oral intake between groups

Variables	Groups		p-value
	ALS	Older adults	
	n = 56 (%)	n = 45 (%)	
Onset of pharyngeal response			
Valleculae	39 (69.6)	39 (86.7)	0.043^*^
Pyriform sinuses	17 (30.4)	6 (13.3)	
YPRSRS	3 (3-3)	2 (1-3)	<0.001^**^
FOIS	5 (4-6)	6 (5-7)	<0.001**

All data are expressed as numbers (%) and medians (interquartile range)

* Pearson's chi-square test;

** Mann-Whitney U test

**Caption:** ALS = amyotrophic lateral sclerosis; YPRSRS = Yale Pharyngeal Residue Severity Rating Scale; FOIS = Functional Oral Intake Scale

The ALS group was subdivided according to the time since diagnosis: approximately 42 participants (75%) had been diagnosed for up to 3 years, while 14 (25%) had been diagnosed for more than 3 years. In the intragroup analysis, there was a significant difference between the onset of pharyngeal response, severity of pharyngeal residues, and level of oral intake, as shown in Table 4. Individuals with more than 3 years of ALS diagnosis presented onset of pharyngeal response in the pyriform sinuses, moderate pharyngeal residues (YPRSRS - 4), and oral intake dependent on an alternative feeding route with consistent oral intake (FOIS 3), compared to individuals with less than 3 years of diagnosis.

**Table 4:** Onset of pharyngeal response, severity of pharyngeal residues, and level of oral intake in relation to time since diagnosis in the group with amyotrophic lateral sclerosis

Variables	Time since diagnosis in the ALS group		p-value
	Up to 3 years	More than 3 years	
	n = 42 (%)	n = 14 (%)	
Onset of pharyngeal response			
Valleculae	35 (83.3)	4 (28.5)	<0.001^*^
Pyriform sinuses	7 (16.6)	10 (71.4)	
YPRSRS	3 (1-3)	4 (3–5)	<0.001^**^
FOIS	5 (5-6)	3 (1.2-4.7)	<0.001**

All data are expressed as numbers (%) or medians (interquartile range)

* Pearson's chi-square test;

** Mann-Whitney U test

**Caption:** ALS = amyotrophic lateral sclerosis; YPRSRS = Yale Pharyngeal Residue Severity Rating Scale; FOIS = Functional Oral Intake Scale

## DISCUSSION

The results of this study showed differences in instrumental swallowing findings in different food consistencies between patients with ALS and older dysphagic adults without neurological diagnosis.

Physiological functions gradually decline in aging due to cellular senescence^(23)^. This deterioration is influenced by cellular and molecular phenomena, which contribute to the increased risk of dysphagia in supposedly healthy older people^(23)^. Conversely, the degenerative ALS process is characterized by muscle weakness, which affects several functions, including swallowing^(24)^. Although swallowing disorders manifest differently between individuals with ALS and older adults, their differences have been evaluated.

Pharyngeal signs of dysphagia were analyzed using FEES with four different food consistencies. The ALS group showed greater swallowing impairment, especially when drinking liquids, than the older adults without a neurological diagnosis. The most common signs were posterior oral leakage, pharyngeal residue, and laryngeal penetration, regardless of whether the liquid was thickened or not.

The frequency of laryngeal penetration in this study’s ALS group was 26% with the thin liquid (level 0), a result very close to the 27% reported in a previous study with the same consistency^(25)^. Meanwhile, the most severe impairment in swallowing safety, laryngotracheal aspiration, was identified in 19.6% of participants in this group. These findings suggest that, compared to older dysphagic adults without a neurological diagnosis, ALS patients were less capable of protecting the lower airway, especially when ingesting less viscous consistencies, due to the particularities of their swallowing disorders.

Because liquids are easy to flow, less viscous, and require greater oral motor control, they can be challenging for patients with ALS^(25,26)^. Tongue motor disorders appear to be the main factors contributing to oropharyngeal dysphagia in the disease, resulting in poor bolus propulsion and delayed onset of the pharyngeal response^(25,27)^. Thus, neuromuscular impairments in the oropharyngeal muscles can affect swallowing efficiency, such as the presence of 75% pharyngeal residue with thin liquid (level 0), which increases the risk of penetration and aspiration^(27)^.

Pharyngeal residues were frequent, persistent, and occurred with all food consistencies evaluated in the ALS group. Most participants with the diagnosis had residues classified as mild, converging with previously published data on neurogenic dysphagia^(28)^. Identifying and classifying residues are important for monitoring disease progression and guiding specific interventions, as they expand the possibilities of therapeutic strategies aimed at increasing swallowing safety and maintaining adequate oral intake.

However, parameters differed between the ALS subgroups divided according to time since diagnosis. Those diagnosed more than 3 years before were more likely to have an early pharyngeal response in the pyriform sinuses, moderate pharyngeal residuals (YPRSRS - 4), and lower levels of oral intake with dependence on alternative feeding routes (FOIS 3). These findings are crucial to understand the rapid progression of the disease and its impact on swallowing function, compared to those without a diagnosis^(26)^. Although nutritional recommendations indicate that enteral feeding can improve survival in ALS patients, other studies have not observed this impact after a prolonged time since diagnosis^(29,30)^.

Patients with ALS often use an alternative feeding route, which is indicated in up to 25.7% of cases each semester^(31)^. The main risk factors associated with using this route are advanced age and prolonged neurodegeneration time since diagnosis^(26)^. The administration of enteral feeding, associated with early detection and appropriate management of oropharyngeal dysphagia, can increase survival by up to 70% of patients with ALS, which reinforces the importance of continuous monitoring of swallowing function^(25,26)^. This is particularly important because recurrent episodes of aspiration increase the risk of hospitalizations for pneumonia, which can compromise the patient's quality of life, nutritional status, and vital capacity^(29)^.

Although the diagnosis of ALS is intrinsically linked to aging^(7,29,30)^, the results of this study differentiate the biomechanical characteristics of swallowing in both cases. Disease progression, as a function of time since diagnosis, has been shown to affect dysphagia-related findings in this population. These factors should be considered in screening, assessment, and intervention to maintain efficient and safe swallowing function and to support new hypotheses for future research.

The limitations of this study include the variability in sample characteristics, such as the number of participants in each group, the different etiopathogenesis of the disease, and the age range. Furthermore, it was not possible to obtain data on symptom onset (limb or bulbar) due to the nature of the outpatient demand, as well as detailed information on medications being used. Regarding the instrumental examination, the evaluators were not blinded to the interpretation of the results, and only one volume was assessed for each food consistency, which may not represent the patient's consumption. Among the strengths are the sample size, the intragroup analysis regarding time since diagnosis, and the longer period dedicated to development and data collection.

## CONCLUSION

The group of individuals with ALS was significantly different from older adults without a diagnosis of neurological disease in the occurrence of multiple swallows, posterior oral leakage, pharyngeal residues, penetration, and aspiration across three food consistencies. Moreover, the time since diagnosis was a determining factor for the onset of pharyngeal response in pyriform sinuses, the presence of moderate pharyngeal residues, and enteral-dependent oral intake.
