# Supplementary material for: Fiberoptic endoscopic evaluation of swallowing in amyotrophic lateral sclerosis: comparison with older people with dysphagia and relationship with time since diagnosis
Source: Codas. 2025 Nov 17;37(6):e20240296. doi: 10.1590/2317-1782/e20240296en (PMC12642774; doi:10.1590/2317-1782/e20240296en)
Supplement: Translation

# Translation: Videoendoscopia da deglutição na Esclerose Lateral Amiotrófica: comparação com idosos disfágicos e relação com o tempo de diagnóstico

**DOI:** 10.1590/2317-1782/e20240296pt

**Authors:** Ramon Cipriano Pacheco de Araújo, Cynthia Meira de Almeida Godoy, Lidiane Maria de Brito Macedo Ferreira, Juliana Fernandes Godoy, Hipólito Magalhães

**Affiliations:** 1 Programa Associado de Pós-graduação em Fonoaudiologia – PPGFON, Universidade Federal do Rio Grande do Norte – UFRN - Natal (RN), Brasil.; 2 Hospital Universitário Onofre Lopes – HUOL - Natal (RN), Brasil.; 3 Departamento de Fonoaudiologia, Universidade Federal do Rio Grande do Norte – UFRN - Natal (RN), Brasil.

**Keywords:** Esclerose Lateral Amiotrófica, Transtornos de Deglutição, Deglutição, Idoso, Faringe

## Abstract

**Objetivo:**

(1) comparar os achados da avaliação instrumental da deglutição entre indivíduos com Esclerose Lateral Amiotrófica (ELA) e idosos disfágicos sem diagnóstico neurológico; (2) comparar o início de resposta faríngea, resíduos faríngeos e o nível de ingestão oral em relação ao tempo de diagnóstico no grupo com ELA.

**Método:**

Trata-se de um estudo transversal e retrospectivo com coleta de dados nos prontuários. Foram incluídos 101 indivíduos com disfagia, estratificados em dois grupos: o primeiro foi composto por 56 pacientes com diagnóstico de ELA e o segundo por 45 idosos. Os sinais de disfagia foram analisados por meio da videoendoscopia da deglutição, utilizando quatro níveis de consistência alimentar, classificados pelo International Dysphagia Diet Standardisation Initiative (IDDSI). Os resíduos faríngeos foram classificados pela Yale Pharyngeal Residue Severity Rating Scale (YPRSRS), e a ingestão oral, pela Functional Oral Intake Scale (FOIS).

**Resultados:**

O grupo com ELA apresentou diferenças em relação às deglutições múltiplas em um nível; escape oral posterior, resíduos faríngeos e penetração laríngea em três níveis; e aspiração em um nível do IDDSI. Os indivíduos com mais de três anos de diagnóstico apresentaram diferenças no início da resposta faríngea nos seios piriformes, resíduos faríngeos moderados e no nível de ingestão oral.

**Conclusão:**

O grupo de indivíduos com ELA apresentou diferenças significativas na ocorrência de deglutições múltiplas, escape oral posterior, resíduos faríngeos, penetração e aspiração em três níveis do IDDSI. Além disso, o tempo de diagnóstico foi um fator determinante para os três parâmetros analisados.

## INTRODUÇÃO

A Esclerose Lateral Amiotrófica (ELA) é uma doença neurodegenerativa progressiva que afeta os neurônios motores superiores e inferiores, frequentemente manifestando-se na fase adulta^(1)^. Sua etiopatogenia envolve a degeneração do sistema motor em diferentes níveis, como bulbar, cervical, torácico e apendicular, resultando em impactos severos na qualidade de vida, especialmente na funcionalidade e na capacidade motora^(2)^. Nos estágios iniciais, um dos sintomas mais comuns é a dificuldade na deglutição, que progride de forma debilitante^(3)^. Com o avanço da doença, complicações respiratórias, como a pneumonia por aspiração, decorrente de uma disfagia orofaríngea grave, podem ser a causa de óbito^(4)^.

Em indivíduos com ELA, a disfagia pode afetar todas as fases da deglutição, evoluindo para a incapacidade completa de ingestão oral^(2)^. Evidências indicam um enfraquecimento progressivo da musculatura orofaríngea, em especial da língua, além de redução do fluxo aéreo expiratório, como o fluxo de tosse, essencial para a proteção das vias aéreas inferiores após a deglutição^(5)^. Aproximadamente 30% dos pacientes com sintomas bulbares desenvolvem alterações na fala e na deglutição nas fases iniciais da doença, com piora progressiva ao longo da evolução clínica^(6)^. Idade avançada e maior tempo de diagnóstico estão associados a quadros mais graves de disfagia^(7)^.

O processo de envelhecimento está associado a diversas modificações fisiológicas, incluindo deterioração neurológica e perda de massa muscular em regiões específicas, com impacto negativo no processo de deglutição^(8)^. A diminuição da massa muscular, da força e da elasticidade dos tecidos conjuntivos orofaríngeos resulta em menor eficiência funcional da língua, dos lábios e da mandíbula^(9)^. Em indivíduos idosos, observa-se acúmulo de tecido adiposo e aumento na deposição de tecido conjuntivo^(8)^. De modo geral, essas modificações contribuem para a redução da eficácia dos mecanismos de mastigação e deglutição^(10)^. A diminuição da elasticidade dos tecidos conjuntivos pode restringir a movimentação anterior da laringe, elevando o risco de resíduos faríngeos após a deglutição^(11)^. Tais alterações, intrinsecamente relacionadas ao envelhecimento, aumentam a suscetibilidade à disfagia^(10,11)^. Além disso, fatores adicionais de estresse fisiológico, como hospitalizações ou efeitos adversos de fármacos, podem comprometer ainda mais o equilíbrio entre as alterações da deglutição e a capacidade compensatória do indivíduo^(11)^.

De acordo com dados epidemiológicos atuais, a idade média de início da ELA esporádica é de aproximadamente 60 anos^(12)^. No entanto, diversos estudos de base populacional têm relatado um aumento progressivo nesta idade média de início, como observado na Europa, onde a média é de 63,7 anos^(13,14)^. Além disso, há evidências crescentes de um aumento na taxa de incidência da ELA com o avanço da idade, com maior prevalência em indivíduos mais velhos^(15)^. Considerando que esses achados refletem uma maior concentração da doença na população idosa, intensifica-se o interesse em investigar e identificar possíveis diferenças na fisiopatologia da deglutição entre indivíduos portadores de ELA e idosos disfágicos sem diagnóstico neurológico.

Portanto, os objetivos deste estudo foram: comparar os parâmetros da deglutição entre indivíduos com ELA e idosos sem diagnóstico neurológico; comparar o início de resposta faríngea, resíduos faríngeos e o nível de ingestão oral em relação ao tempo de diagnóstico no grupo com ELA.

## MÉTODO

Trata-se de um estudo transversal e retrospectivo com base na coleta de dados nos prontuários. A pesquisa foi conduzida no ambulatório de otorrinolaringologia do Hospital Universitário Onofre Lopes, Universidade Federal do Rio Grande do Norte, Natal (RN), em que foram coletados dados referentes ao exame da videoendoscopia da deglutição (VED) dos pacientes atendidos entre 2017 e 2023. Todos os participantes, ou responsáveis legais, assinaram o Termo de Consentimento Livre e Esclarecido (TCLE) disponibilizado previamente pelo serviço antes da realização dos procedimentos do exame. O estudo foi aprovado pelo Comitê de Ética em Pesquisa do Hospital Universitário Onofre Lopes - CEP, sob parecer n° 6.169.294. Os dados coletados foram referentes aos achados do exame instrumental e o nível de ingestão por via oral após o exame.

### Casuística

A amostra foi constituída por 101 pacientes disfágicos escolhidos por conveniência entre os indivíduos que buscaram atendimento no referido local. Para o primeiro objetivo, os participantes foram divididos em dois grupos de acordo com a presença do diagnóstico de ELA. O primeiro grupo foi composto por 56 indivíduos com ELA independente do tempo de diagnóstico, idade entre 39 e 83 anos e com predomínio do gênero masculino (57,1%). O segundo grupo foi composto por 45 idosos, com idade entre 60 e 90 anos, que apresentavam queixas clínicas de disfagia, sem diagnóstico de doença neurológica, com predomínio do gênero feminino (57,7%).

Para o segundo objetivo do estudo, o grupo com ELA foi subdividido em dois subgrupos: “até 3 anos” e “mais de 3 anos” de diagnóstico, para análise intragrupo. O ponto de corte de 3 anos foi definido com base na literatura, que indica esse período como a média para a introdução de alimentação enteral nessa população^(16)^.

Os critérios de exclusão para ambos os grupos foram: (a) outros diagnósticos neurológicos agudos ou progressivos; (b) incapazes de seguir comandos; (c) histórico de tratamento oncológico de cabeça e pescoço; (d) usuários de traqueostomia; (e) histórico de intubação orotraqueal nos últimos 12 meses prévios ao exame.

Destacamos que todos os participantes da pesquisa apresentavam queixas de deglutição e estavam em processo de investigação da disfagia, identificados por outros profissionais de saúde e/ou encaminhados por outros setores do próprio hospital em interconsultas.

### Procedimentos

O exame da videoendoscopia da deglutição foi realizado por um médico residente, acompanhado por um otorrinolaringologista e um fonoaudiólogo com experiência em disfagia orofaríngea, em consonância com o protocolo da instituição. Foi utilizado um nasofibroscópio flexível, marca Olympus® de 3,2mm de diâmetro, modelo LF-P com microcâmera e fonte de luz acoplados. O paciente foi orientado a permanecer na posição sentada e tronco ereto durante toda realização do exame, não sendo utilizado anestésico tópico durante a introdução do instrumento na cavidade nasal até a região de hipofaringe.

Durante a inspeção das estruturas, a sensibilidade faríngea foi avaliada por meio de dois toques do nasofibroscópio na região da epiglote, que foi observado a presença de reação de constrição faríngea. O fechamento glótico foi analisado solicitando ao paciente a emissão prolongada da vogal “i”. Considerou-se um fechamento reduzido quando as pregas vocais coaptaram em menos de 2/3 da sua extensão^(17)^.

O fonoaudiólogo foi responsável por ofertar os líquidos, sem adição de sabores artificiais, corados artificialmente com anilina azul e espessados por meio de um produto instantâneo a base de amido de milho. Ao final, foi ofertado também uma porção de biscoito salgado de 8 g por demanda livre.

As consistências alimentares foram classificadas a partir do *International Dysphagia Diet Standardisation Initiative* (IDDSI)^(18)^ e seguiram a ordem de oferta: nível 2 (líquido levemente espessado), nível 4 (líquido extremamente espessado) e nível 0 (líquido fino) em três ofertas com uma colher metálica de 5 mL, enquanto o nível 7 (sólido regular) foi uma porção única.

Os três profissionais anteriormente citados, com experiência na realização do exame, foram responsáveis por interpretar e avaliar simultaneamente, por consenso, a presença de deglutições múltiplas, escape oral posterior, resíduos faríngeos em região de valéculas e/ou seios piriformes, de acordo com a classificação *Yale Pharyngeal Residue Severity Rating Scale* (YPRSRS)^(19)^ (1 - Nenhum, 2 - Vestígio faríngeo, 3 - Resíduo leve, 4 - Resíduo moderado, 5 - Resíduo grave), além de avaliar a penetração laríngea e a aspiração laringotraqueal.

Para a análise, foram considerados os seguintes parâmetros, com base no pior desempenho observado a partir da primeira oferta: deglutições múltiplas, considerada como mais de duas tentativas de deglutir a mesma oferta^(20)^; escape oral posterior, pela presença do escape prematuro do alimento em região de hipofaringe antes de desencadear a reação de deglutição^(20)^; resíduo faríngeo, por meio da identificação de presença residual de alimento corado em região de valéculas e/ou seios piriformes, após a deglutição da primeira oferta em diante^(19)^; penetração laríngea, via observação de presença residual de alimento corado em região de pregas vocais^(21)^; e aspiração laringotraqueal, quando houvesse resíduo de alimento corado abaixo das pregas vocais^(21)^. Toda análise aconteceu em tempo real e as imagens foram armazenadas em um computador do próprio ambulatório, para serem revisadas quantas vezes os profissionais julgassem necessárias após a realização do exame.

Para determinar a ingestão por via oral foi aplicado o *Functional Oral Intake Scale* (FOIS)^(22)^ após a realização do exame. Os três profissionais consideraram os achados instrumentais, o histórico clínico, a existência e a necessidade de espessamento dos líquidos. As pontuações na escala FOIS variam de 1 (nada por via oral) a 7 (ingestão total por via oral sem restrições).

### Análise dos dados

Para análise dos dados, foi utilizada a estatística descritiva e inferencial por meio de medidas de tendência central e de dispersão, além de frequências absolutas e relativas. Foi aplicado o teste de normalidade Shapiro-Wilk para verificar a distribuição entre as variáveis quantitativas dependentes do estudo. Para análise inferencial, foi aplicado o Qui-quadrado de Pearson ou Exato de Fisher para analisar as variáveis categóricas, a depender da frequência esperada de cada célula ser superior ou igual a 5. Enquanto o teste t de Student foi utilizado para analisar as variáveis numéricas. Para análise e comparação dos protocolos utilizados, foi aplicado o teste U de Mann-Whitney após verificação da distribuição da normalidade. Para todas as análises considerou-se o nível de significância de 5%.

## RESULTADOS

A amostra foi constituída por 101 indivíduos disfágicos divididos em dois grupos de acordo com a presença do diagnóstico de ELA. O primeiro grupo foi formado por 56 indivíduos com diagnóstico de ELA, sendo 83,9% de forma esporádica e 16,0% de forma familiar. Enquanto o segundo grupo foi formado por 45 idosos disfágicos sem diagnóstico neurológico.

Entre as pessoas idosas, cerca de 40% apresentavam comorbidades como diabetes mellitus, 24,4% doenças cardiovasculares, 15,5% doenças digestivas e 8,8% transtornos depressivos. Nenhum participante realizou terapia fonoaudiológica anteriormente. Na Tabela 1 constam a análise comparativa entre gênero e idade entre os grupos.

**Tabela 1:** Análise comparativa entre gênero e idade entre os grupos

**Variáveis**	**Grupos**		**Valor de *p***
	ELA	Idosos	
	n = 56 (%)	n = 45 (%)	
Gênero			
Masculino	32 (57,1)	19 (42,2)	0,136
Feminino	24 (42,9)	26 (57,8)	
Idade (anos)	62,1 (± 11,4)	71,0 (± 7,72)	**<0,001** ^*^

* Teste t de Student

Legenda: ELA = Esclerose Lateral Amiotrófica

A Tabela 2 compara os achados do exame instrumental por nível de consistência alimentar entre os grupos. Houve diferença significativa entre todos os achados na consistência de líquido fino (nível 0) e entre escape oral posterior, resíduos faríngeos e penetração laríngea nas consistências de líquido levemente espessado (nível 2) e líquido extremamente espessado (nível 4). Não foram observadas diferenças na consistência de sólido regular (nível 7) entre os grupos.

**Tabela 2:** Comparação entre os achados videoendoscópios por nível de consistência alimentar entre os grupos

Achados videoendoscópios por nível de consistência alimentar (IDDSI)	Grupos		Valor de *p*
	ELA	Idosos	
	n = 56 (%)	n = 45 (%)	
Sensibilidade faríngea			
Preservada	48 (85,7)	35 (77,8)	0,3
Reduzida	8 (14,3)	10 (22,2)	
Fechamento glótico			
Preservada	53 (94,6)	44 (97,8)	0,422
Reduzida	3 (5,4)	1 (2,2)	
Líquido fino (nível 0)			
Deglutições múltiplas			
Ausente	46 (82,1)	43 (95,6)	**0,038** ^**^
Presente	10 (17,9)	2 (4,4)	
Escape oral posterior			
Ausente	20 (35,7)	27 (60,0)	**0,015** ^*^
Presente	36 (64,3)	18 (40,0)	
Resíduos faríngeos			
Ausente	14 (25,0)	25 (55,6)	0,002*
Presente	42 (75,0)	20 (44,4)	
Penetração laríngea			
Ausente	41 (73,2)	44 (97,8)	<0,001**
Presente	15 (26,8)	1 (2,2)	
Aspiração laringotraqueal			
Ausente	45 (80,4)	44 (97,8)	0,011**
Presente	11 (19,6)	1 (2,2)	
Líquido levemente espessado (nível 2)			
Deglutições múltiplas			
Ausente	48 (85,7)	43 (95,6)	0,178
Presente	8 (14,3)	2 (4,4)	
Escape oral posterior			
Ausente	26 (46,4)	34 (75,6)	0,003*
Presente	30 (53,6)	11 (24,4)	
Resíduos faríngeos			
Ausente	16 (28,6)	26 (57,8)	0,003*
Presente	40 (71,4)	19 (42,2)	
Penetração laríngea			
Ausente	46 (82,1)	44 (97,8)	0,021**
Presente	10 (17,9)	1 (2,2)	
Aspiração laringotraqueal			
Ausente	51 (91,1)	45 (100)	0,064
Presente	5 (8,9)	0 (0,0)	
Líquido extremamente espessado (nível 4)			
Deglutições múltiplas			
Ausente	46 (82,1)	43 (95,6)	0,061
Presente	10 (17,9)	2 (4,4)	
Escape oral posterior			
Ausente	27 (48,2)	31 (68,9)	0,037*
Presente	29 (51,8)	14 (31,1)	
Resíduos faríngeos			
Ausente	18 (32,1)	25 (55,6)	0,018*
Presente	38 (67,9)	20 (44,4)	
Penetração laríngea			
Ausente	50 (89,3)	45 (100)	0,032**
Presente	6 (10,7)	0 (0,0)	
Aspiração laringotraqueal			
Ausente	53 (94,6)	45 (100)	0,251
Presente	3 (5,4)	0 (0,0)	
Sólido regular (nível 7)			
Deglutições múltiplas			
Ausente	53 (94,6)	45 (100)	0,251
Presente	3 (5,4)	0 (0,0)	
Escape oral posterior			
Ausente	49 (87,5)	40 (88,9)	0,83
Presente	7 (12,5)	5 (11,1)	
Resíduos faríngeos			
Ausente	40 (71,4)	39 (86,7)	0,065
Presente	16 (28,6)	6 (13,3)	
Penetração laríngea			
Ausente	56 (100)	45 (100)	-
Presente	0 (0,0)	0 (0,0)	
Aspiração laringotraqueal			
Ausente	56 (100)	45 (100)	-
Presente	0 (0,0)	0 (0,0)	

Todos os dados foram expressos em números (%)

* Qui-quadrado de Pearson;

** Exato de Fisher

**Legenda:** ELA = Esclerose Lateral Amiotrófica; IDDSI = *International Dysphagia Diet Standardisation Initiative*

Além disso, houve diferença significativa no início de resposta faríngea, na gravidade dos resíduos faríngeos e no nível de ingestão oral entre os grupos apresentados na Tabela 3. Em sua maioria, o grupo com ELA apresentou início de resposta faríngea em valéculas, resíduos faríngeos classificados em grau leve (YPRSRS - 3) e ingestão por via oral total com múltiplas consistências, porém com necessidade de preparo especial ou compensações (FOIS 5).

**Tabela 3:** Comparação entre início de resposta faríngea, gravidade dos resíduos faríngeos e ingestão por via oral entre os grupos

**Variáveis**	**Grupos**		**Valor de *p***
	**ELA**	**Idosos**	
	n = 56 (%)	n = 45 (%)	
Início de resposta faríngea			
Valéculas	39 (69,6)	39 (86,7)	**0,043** ^*^
Seios piriformes	17 (30,4)	6 (13,3)	
YPRSRS	3 (3-3)	2 (1-3)	**<0,001** ^**^
FOIS	5 (4-6)	6 (5-7)	<0,001**

Todos os dados foram expressos em números (%) e mediana (intervalo interquartil 25-75).

* Qui-quadrado de Pearson;

** U de Mann-Whitney

Legenda: ELA = Esclerose Lateral Amiotrófica; YPRSRS = *Yale Pharyngeal Residue Severity Rating Scale*; FOIS = *Functional Oral Intake Scale*

O grupo com ELA foi subdividido de acordo com o tempo de diagnóstico: cerca de 42 participantes (75%) apresentavam até 3 anos de diagnóstico, enquanto 14 (25%) tinham mais de 3 anos de diagnóstico. Na análise intragrupo, houve diferença significativa entre início de resposta faríngea, gravidade dos resíduos faríngeos e nível de ingestão oral apresentados na Tabela 4. Os indivíduos com mais de 3 anos do diagnóstico de ELA apresentaram início de resposta faríngea em seios piriformes, resíduos faríngeos em grau moderado (YPRSRS - 4) e ingestão oral dependente de via alternativa de alimentação com consistente via oral (FOIS 3), em comparação aos indivíduos com menos de 3 anos de diagnóstico.

**Tabela 4:** Início de resposta faríngea, gravidade dos resíduos faríngeos e nível de ingestão oral em relação ao tempo de diagnóstico no grupo com ELA

Variáveis	Tempo de diagnóstico no grupo com ELA		**Valor de *p***
	Até 3 anos	Mais de 3 anos	
	n = 42 (%)	n = 14 (%)	
Início de resposta faríngea			
Valéculas	35 (83,3)	4 (28,5)	**<0,001** ^*^
Seios piriformes	7 (16,6)	10 (71,4)	
YPRSRS	3 (1-3)	4 (3–5)	<0,001**
FOIS	5 (5-6)	3 (1,2-4,7)	**<0,001** ^**^

Todos os dados são expressos em números (%) ou mediana (intervalo interquartil 25-75)

* Qui-quadrado de Pearson;

** U de Mann-Whitney

**Legenda:** ELA = Esclerose Lateral Amiotrófica; YPRSRS = *Yale Pharyngeal Residue Severity Rating Scale*; FOIS = *Functional Oral Intake Scale*

## DISCUSSÃO

Os resultados deste estudo mostraram diferenças nos achados instrumentais da deglutição em diferentes consistências alimentares entre pacientes com ELA e idosos disfágicos sem diagnóstico neurológico.

Durante o envelhecimento, as funções fisiológicas diminuem gradualmente à medida que ocorre a senescência celular^(23)^. Esse processo de deterioração é influenciado por fenômenos celulares e moleculares, que contribuem para o aumento do risco de disfagia em idosos supostamente saudáveis^(23)^. Por outro lado, o processo degenerativo da esclerose lateral amiotrófica é caracterizado pela fraqueza muscular, que afeta várias funções, incluindo a deglutição^(24)^. Embora os transtornos de deglutição se manifestem de formas distintas entre indivíduos com ELA e idosos, suas diferenças foram avaliadas.

Os sinais faríngeos da disfagia foram analisados por meio da VED com quatro consistências alimentares diferentes. O grupo com ELA apresentou maior comprometimento da deglutição, especialmente na ingestão de líquidos, em comparação aos idosos sem diagnóstico neurológico. Os sinais mais frequentes foram o escape oral posterior, resíduos faríngeos e penetração laríngea, independente do líquido estar espessado ou não.

No grupo com ELA, a frequência da penetração laríngea observada neste estudo foi de 26% com o líquido fino (nível 0), um resultado muito próximo dos 27% relatados em um estudo anterior com a mesma consistência^(25)^. Enquanto o comprometimento mais grave na segurança da deglutição, a aspiração laringotraqueal, foi identificado em 19,6% dos participantes desse grupo. Esses achados sugerem que, em comparação com idosos disfágicos sem diagnóstico neurológico, pacientes com ELA apresentaram menor capacidade de proteger as vias aéreas inferiores, especialmente ao ingerir consistências menos viscosas, devido às particularidades de seus transtornos de deglutição.

Em razão dos líquidos serem fáceis de fluir, menos viscosos e necessitarem de um controle motor oral maior, esses podem ser desafiadores para o paciente com ELA^(25,26)^. Os distúrbios motores da língua parecem ser os principais fatores que contribuem para a disfagia orofaríngea na doença, na qual resultam em uma fraca propulsão do bolo alimentar e atraso no início de resposta faríngea^(25,27)^. Assim, alterações neuromusculares na musculatura orofaríngea podem ocasionar alterações na eficiência da deglutição, como a presença de 75% de resíduos faríngeos em líquido fino (nível 0), o que aumenta o risco de penetração e aspiração^(27)^.

Os resíduos faríngeos foram frequentes, persistentes e ocorreram em todas as consistências alimentares avaliadas no grupo com ELA. À vista disso, os participantes com o diagnóstico apresentaram, em sua maioria, resíduos classificados em grau leve, convergente com dados previamente publicados nas disfagias neurogênicas^(28)^. A identificação e a classificação dos resíduos são importantes para monitorar a progressão da doença e orientar intervenções específicas, pois ampliam as possibilidades de estratégias terapêuticas que visam aumentar a segurança da deglutição e manter a ingestão oral adequada.

No entanto, ao dividirmos o grupo com ELA de acordo com o tempo de diagnóstico, houve diferenças nos parâmetros analisados. Aqueles que apresentavam mais de 3 anos de diagnóstico foram preponderantes em início de resposta faríngea em seios piriformes, resíduos faríngeos em grau moderado (YPRSRS - 4) e menor nível de ingestão oral com dependência de via alternativa de alimentação (FOIS 3). Tais achados são de fundamental compreensão no que diz respeito a rápida progressão da doença e seu impacto na função de deglutição, em comparação àqueles sem o diagnóstico^(26)^. Embora as recomendações nutricionais indiquem que o uso da alimentação enteral possa melhorar a sobrevida de pacientes com ELA, outros estudos não observaram esse impacto após um tempo prolongado do diagnóstico^(29,30)^.

O uso de via alternativa de alimentação em pacientes com ELA é frequente, sendo indicada em até 25,7% dos casos a cada semestre^(31)^. Os principais fatores de risco associados ao uso dessa via são a idade avançada e o tempo prolongado da neurodegeneração a partir do diagnóstico^(26)^. A administração da alimentação enteral, associada à detecção precoce e ao manejo adequado da disfagia orofaríngea, pode aumentar a sobrevida em até 70% dos pacientes com ELA, o que reforça a importância do acompanhamento contínuo da funcionalidade da deglutição^(25,26)^. Isso é particularmente importante, pois episódios recorrentes de aspiração aumentam o risco de hospitalizações por pneumonia, o que pode comprometer a qualidade de vida, o estado nutricional e a capacidade vital do paciente^(29)^.

Apesar do diagnóstico de ELA estar intrinsecamente ligado ao envelhecimento^(7,29,30)^, os resultados do presente estudo conseguem diferenciar as características da biomecânica da deglutição em ambos os casos. A progressão da doença, em função do tempo de diagnóstico, demonstrou interferir nos achados relacionados à disfagia nessa população. Esses fatores devem ser considerados no rastreamento, avaliação e intervenção, visando manter a funcionalidade da deglutição de forma eficiente e segura, além de subsidiar novas hipóteses para pesquisas futuras.

Entre as limitações deste estudo, destacam-se a variabilidade nas características da amostra, como o número de participantes para cada grupo, diferentes etiopatogenias da doença e faixa etária. Além disso, não foi possível obter dados sobre o início dos sintomas (apendicular ou bulbar) devido à natureza da demanda ambulatorial, bem como informações detalhadas sobre os medicamentos em uso. No que se refere ao exame instrumental, os avaliadores não foram cegos quanto à interpretação dos resultados, e foi avaliado apenas um volume para cada consistência alimentar, o que pode não representar o consumo do paciente. Entre os pontos fortes, destacam-se o tamanho da amostra, a análise intragrupo em relação ao tempo de diagnóstico e o período mais longo dedicado ao desenvolvimento e à coleta de dados.

## CONCLUSÃO

O grupo de indivíduos com esclerose lateral amiotrófica apresentou diferenças significativas em relação aos idosos sem diagnóstico de doença neurológica na ocorrência de deglutições múltiplas, escape oral posterior, resíduos faríngeos, penetração e aspiração em três níveis de consistência alimentar. Além disso, o tempo de diagnóstico foi um fator determinante para o início da resposta faríngea em seios piriformes, presença de resíduos faríngeos em grau moderado e a ingestão oral dependente de via enteral.
